# Measuring the associations between brain morphometry and polygenic risk scores for substance use disorders in drug-naive adolescents

**DOI:** 10.21203/rs.3.rs-6190536/v1

**Published:** 2025-04-01

**Authors:** Sydney Kramer, Mei-Hsin Su, Mallory Stephenson, Jill Rabinowitz, Brion Maher, Roxann Roberson-Nay, Luis F.S. Castro-de-Arajuo, Yi Zhou, Michael C. Neale, Nathan Gillespie

## Abstract

Substance use has been associated with differences in adult brain morphology; however, it is unclear whether these differences precede or are a result of substance use substance use. We investigated the impact of polygenic risk scores (PRSs) for cannabis use disorder (CUD) and general substance use and substance use disorder liability (SU/SUD) on brain morphology in drug-naïve adolescents. Baseline data were used from 1,874 European-descent participants (ages 9–11) comprising 222, 328 and 387 pairs of MZ twins, DZ twins, and Non-Twin Siblings, respectively, in the Adolescent Brain Cognitive Development Study. We fitted multivariate twin models to estimate the putative effects of CUD, SU/SUD, and brain region-specific PRSs. These models assessed their influence on six subcortical and two cortical phenotypes. PRS for CUD and SU/SUD were created based on GWAS conducted by Johnson et al. (2020) and Hatoum et al. (2023), respectively. When decomposing variance in each brain region of interest (ROI), we used the corresponding ROI-specific PRS. Brain morphometry in drug-naive subjects was unrelated to CUD PRS. The variance explained in each ROI by its corresponding PRS ranged from 0.8–4.4%. The SU/SUD PRS showed marginally significant effects (0.2–0.4%) on cortical surface area and nucleus accumbens volume, but overall effect sizes were small. Our findings indicate that differences in brain morphometry among baseline drug-naive individuals are not associated with the genetic risk for CUD but show a weak association with general addiction and substance use risk (SU/SUD), particularly in nucleus accumbens volume and total cortical surface area.

### Introduction

The use of cannabis and other substances is common in adolescents and young adults (Swendsen et al., 2012; Volkow et al., 2021). However, frequent drug use can be hazardous to mental health (Conway et al., 2018; Hines et al., 2020; Yurasek et al., 2017). During development, there are dynamic changes in brain neurochemistry, fiber architecture, and tissue composition (Bava & Tapert, 2010). These changes may be impacted by chronic substance use (SU) (Battistella et al.,

2014; Gray & Squeglia, 2018). Indeed, reports based on large samples of adults have shown that SU is associated with variations in brain morphometry (Gillespie et al., 2018; Lange et al., 2017, p. 20; Paul & Bhattacharyya, 2018; Rabinowitz et al., 2022). Given changing cultural norms and expanding cannabis medicalization and decriminalization, identifying the effects of cannabis use (CU) on brain morphology specifically, and comorbid SU more broadly, is critical.

There is increasing agreement that phenotypic SU and substance use disorders (SUDs) associate with structural brain differences. Alcohol, tobacco, and cannabis use have been linked to such differences based on findings from large cross-sectional and meta-analyses of adult samples(Gillespie et al., 2018; Lange et al., 2017; Paul & Bhattacharyya, 2018; Rabinowitz et al., 2022). A multinational study involving 2,277 SUD cases and 1628 controls (mean ages 2739) found reduced radial distance (i.e., local thickness) and Jacobian determinant (i.e., surface area dilation or contraction), reflecting lower thickness and surface area in subcortical structures such as the hippocampus, thalamus, putamen, and amygdala, particularly in individuals with alcohol dependence (Chye et al., 2020). Previous research has suggested non-linear or multivariate patterns between substance use and brain regions (Mackey et al., 2018). The Jacobian determinant was used to map brain region surfaces to a master template, enabling multivariate analysis of substance-related shape variations (Chye et al., 2020); findings from this study found global differences in dependent substance users and controls across subcortical structures. Whether SUD directly causes these differences, or if they correlate with SUD risk for other reasons, remains unclear. Addressing this issue partly motivates the present article.

Navarri et al. (2022) conducted a meta-analysis in ENIGMA with 435 SUD cases and 363 controls (ages 12–60) and found that alcohol use disorder (AUD) associates with reduced volumes in the thalamus, hippocampus, amygdala, and nucleus accumbens, as well as decreased thickness in several cortical areas (e.g., fusiform gyrus, temporal gyrus, and anterior cingulate). Harper et al. (2021) studied 436 twins, and found that both alcohol and cannabis use were associated with reduced cortical thickness in the orbitofrontal cortices. Gillespie et al. (2018) reported a significant association between nicotine use and thalamus volume in a U.S. sample (N = 474, mean age 56.1) but no association for cannabis use with other subcortical regions. This may suggest that either larger samples are needed or normal cannabis use does not correlate with brain morphology. Furthermore, Prom-Wormley et al. (2015) found associations between average lifetime cigarette use and brain structure, with modestly reduced volume and surface area among cigarette smokers. Given that cannabis use is strongly comorbid with both tobacco and alcohol use, it is challenging to identify effects of cannabis use per se. Lastly, Miller et al. (2024) observed an association between early initiation of any substance in adolescents and greater whole brain, cortical, and subcortical volumes, and thinner prefrontal cortex volumes. However, these associations may reflect individual variability in predisposition to risk-taking and substance use. Nevertheless, there does appear to be an emerging consensus that substance use is related to individual differences in brain morphology. However, the above reports invariably relied upon samples assessed during or after the mean age of substance use initiation (Chen et al., 2017; Lipari et al., 2017).

Whether the observed differences in brain morphometry preceded or occurred following SU exposure or repeated use is currently unclear. Albaugh et al. (2021) analyzed N = 799 cannabisnaive participants aged ~ 14 years at a baseline assessment and at a five-year follow-up; they found cannabis users exhibited accelerated age-related cortical thinning in the superior and anterior medial prefrontal cortex, with the degree of thinning showing a dose-dependent relationship (Albaugh et al., 2021).While these findings are consistent with changes in brain morphometry arising from exposure to drugs, the authors did not control for genetic or environmental confounding. Therefore, it is possible that any observed impact of SU on brain morphometry arose from non-causal mechanisms such as horizontal pleiotropy, e.g., individuals at higher genetic risk of cannabis use and misuse were predisposed to faster cortical thinning. Resolving these alternative hypotheses requires a genetically informative longitudinal study of initially drug-naive subjects to determine whether or not genetic factors associated with SU predict variation in brain morphometry in drug-naive subjects.

Previous research has demonstrated that genetic predisposition to substance use behaviors can predict brain structure in drug-naive adolescents. Rabinowitz et al. (2022) examined the covariance between i) individual differences in brain morphometry in various regions of interest (ROI), ii) polygenic risk scores (PRS) for five SU classes, and iii) PRSs associated with each ROI (Rabinowitz et al., 2022) in participants at baseline (9–10 years old). They reported higher alcohol use genetic liability predicted lower postcentral gyrus and cortical surface areas, and also observed similar negative associations between a PRS for regular smoking and two ROIs, including intracranial volume and surface area of the inferior temporal gyrus. Thus, genetic risks associated with substance use appear to predict individual differences in brain morphometry prior to exposure. However, the relative contributions of the SU and ROI PRSs are not clear within the context of genetic and environmental sources of variance and covariances, which may be estimated with a suitable research design.

Building upon Rabinowitz et al. (2022), we expanded their approach by investigating both polygenic risk scores (PRS) for substance use disorders (SUDs) and their combined effects on regions of interest (ROIs) in the brain. We are unaware of genetically informative studies in drug-naive adolescents that have addressed the above limitations while resolving the question of whether or not individual differences that have been associated with SU or SUDs were present prior to exposure. Our aims, therefore, are i) to model the joint impacts of PRSs for SUDs and ROI-specific PRSs on a number of putative ROIs, while ii) accounting for genetic correlations between the PRSs and with residual genetic and environmental influences. To achieve these aims, we leveraged data from the Adolescent Brain Cognitive Development (ABCD) study that includes twin pair adolescents who are drug-naive (ages 9–11). We hypothesized that higher polygenic risk scores for CUD and SU/SUD would predict reduced volumes in six putative ROIs and reduced cortical surface area and thickness. However, given the prevalence of comorbid substance use and misuse and significant pleiotropy (Kendler et al., 2000), we hypothesized that a PRS for SU/SUD based on Hatoum et al.'s (2023) GWAS meta-analysis would also predict variation in brain volume in drug-naive subjects. Accordingly, we model data from twin and sibling pairs in the ABCD Study®, which features data on substance use, genotypes from the Smokescreen array suitable for PRS calculations, and structural and functional neuroimaging.

### Methods

## Sample

Data were obtained from sibling pairs (including twins) aged 9–11 years participating in the

Adolescent Brain Cognitive Development (ABCD) study conducted by the National Institute on Drug Abuse. Spanning 22 sites across the United States, the ABCD study is an ongoing longitudinal investigation of child health and development utilizing a nationally representative cohort. A comprehensive description of the range of measures administered as part of the study can be found elsewhere (Volkow et al., 2018). The ABCD cohort comprises N = 11,666 individuals. This analysis focused on a subset of European twin and sibling pairs due to the lack of GWAS on brain morphometry in other ancestries. This subset amounted to N = 1,874 individuals.

### Genotyping

Subjects provided saliva and/or blood samples for DNA extraction during their study visit, either by spitting into small tubes or through a blood draw. DNA extraction and genotyping were conducted by the former Rutgers University Cell and DNA Repository (now known as SAMPLED). Genotyping was performed using the Affymetrix Axiom Smokescreen Array, comprising 646,247 markers, including genome-wide association markers, tag SNPs, exonic markers in addiction-related gene regions, fine-mapping markers in loci related to nicotine metabolism, smoking behavior, and comorbidity markers (Baurley et al., 2016). The array design encompassed nearly all variants in the 1000 Genomes Project Phase 1, NHLBI GO Exome Sequencing Project, and HapMap databases pertinent to smoking behavior and nicotine metabolism. Additionally, the array encompassed genome-wide common variation (65.67%, 82.37%, and 90.72% in African (YRI), East Asian (ASN), and European (EUR) populations respectively) (Baurley et al., 2016).

The Affymetrix Power Tools and the Affymetrix Best Practice Workflow were employed for calling and aligning genotypic data using human genome build hg19 (Baurley et al., 2016; Fan et al., 2023). Batch-level quality control of blood and saliva samples, also performed using these tools, resulted in approximately 590K variants per batch. Samples with more than 20% missing rates on genotype calls or variants with more than 10% missingness were excluded from downstream analyses (Anderson et al., 2010; Fan et al., 2023). Additionally, samples with excessive relatedness or implausible numbers of third-degree relatives were excluded. SNPs were phased and imputed using SHAPEIT2 and IMPUTE2. Following the ABCD study's quality control pipeline (Anderson et al., 2010; Fan et al., 2023), the standard step of sex-genotype concordance checking was intentionally not performed, as the study investigators determined that comparing genotyped sex with reported sex/gender information could mischaracterize participants' identities and raise complex interpretational issues regarding biological sex versus gender identity. After quality control procedures, N = 11,389 unique participants and 516,598 genetic variants remained for analysis (Fan et al., 2023). Genetic principal components were calculated using the R-based software PC-AiR, resulting in 158,103 SNPs after pruning. PC-AiR was then run on this pruned set of SNPs, projecting N = 8,005 unrelated individuals and N = 3,384 related individuals. Additionally, N = 2,504 individuals from the 1000 Genomes Project were projected onto the same PC space as the ABCD sample to provide reference points for different ancestral distributions (AFR, African; AMR, Native American; EAS, East Asian; EUR, European; SAS, South Asian). Genetic relatedness independent of any ancestral relatedness was assessed using PC-Relate, employing the same set of pruned SNPs and the first two genetic principal components.

### Genetic Ancestry and Principal Component Analysis

Ancestry assignment was performed using the method described by Peterson et al., (2017) on N = 11,666 individuals. Ancestry outliers, defined as individuals greater than three standard deviations from from the center of their assigned superpopulation in PCA space, were excluded. The numbers assigned to each 1000 Genomes super population was as follows: N = 2,170 to AFR, 2,909 to AMR, 188 to EAS, 5,815 to EUR, and 372 to SAS. Within-ancestry principal component analysis was conducted for EUR individuals using PC-AiR, following the same method used for trans-ancestry PCA in ABCD Release 5.0. Only individuals with

Europeanderived ancestry were selected for the current analyses due to the absence of brain structure GWAS summary statistics from other ancestral groups.

### Post-Imputation QC and SNP Processing

Imputation was performed for individuals with European ancestry using the 1000 Genomes reference panel. Standard GWAS QC was performed with variants with minor allele frequency

(MAF) < 0.001 and variants failing the Hardy-Weinberg test at a significance threshold of $1 \times 10^{10}$ being excluded. After QC, a total of N = 13,549,177 SNPs remained for analysis.

Before using PRS-CS, we screened the input GWAS summary statistics files for duplicate SNPs. Duplicates were only found in the summary statistics from the thalamus volume GWAS (N = 17 duplicate SNPs), which were removed to ensure each SNP was represented only once in the PRS calculation.

### Genome-Wide Association Study (GWAS) Summary Statistics

Polygenic risk scores (PRS) were estimated for each subject, including a CUD PRS and a SU/SUD PRS. For the SU/SUD PRS, summary statistics from Hatoum et al.'s (2023) multivariate meta-analytic GWAS of CUD, problematic alcohol use, problematic tobacco use, and opioid use disorder were utilized. The CUD GWAS summary statistics used in Johnson et al.'s (2020) multivariate meta-analytic GWAS were also employed to generate a separate CUD PRS (Johnson et al., 2020). Full descriptions of the SNP quality control, alignment, ancestry assignment, and imputation procedures for each discovery GWAS are detailed elsewhere (Fan et al., 2023). Cortical and subcortical GWAS summary statistics were utilized to estimate cortical and subcortical PRSs. For the hippocampus, GWAS summary statistics were derived from a meta-analysis of mean bilateral hippocampal volume from the ENIGMA and CHARGE

consortium. Subcortical GWAS summary statistics were obtained from various studies within the CHARGE consortium, ENIGMA consortium, and UK Biobank (Bycroft et al., 2018; Psaty et al., 2009; Thompson et al., 2014).

## Polygenic Risk Score (PRS) Calculation

PRSs were estimated using Polygenic Risk Score-Continuous Shrinkage (PRS-CS) [https://github.com/getian107/PRScs] method. This method utilizes a Bayesian framework to infer posterior effect sizes of SNPs using genome-wide association summary statistics and an external linkage disequilibrium (LD) panel (Ge et al., 2019). The method then applies a continuous shrinkage (CS) prior to SNP effect sizes in order to shrink many small effect sizes toward zero and allow a small subset of variants with stronger signals to retain larger effect sizes, thereby reducing noise and avoiding overfitting. We wanted to model all common variation of general addiction liability, cannabis use disorder, and brain structures, so utilizing PRS-CS was the most appropriate. The 1000 Genomes European (EUR) Phase 3 reference panel, which was provided with the PRS-CS software, was used as the reference panel. Standard GWAS QC was previously performed on the PRS-CS reference panel which overlapped with 1,062,342 SNPs for CUD, 1,060,634 SNPs for cortical surface area, 1,061,323 SNPs for cortical thickness, 1,042,513 SNPs for brainstem, 1,056,811 for hippocampus, 1,042,259 for nucleus accumbens, 1,039,048 for putamen, 1,038,846 for caudate, 1,041,942 for thalamus. PRSs were estimated for: a general addiction factor (Hatoum et al., 2023); cannabis use disorder (Johnson et al., 2020); cortical surface area and cortical thickness (Grasby et al., 2020); hippocampus, nucleus accumbens, caudate nucleus, putamen and thalamus (Satizabal et al., 2019). Each set of summary statistics was checked for duplicates, and 17 duplicate SNPs were found in the thalamus summary statistics. Uncorrected associations between the eight brain PRSs and their corresponding brain regions are shown in Table 1. Prior to model fitting, all PRSs were standardized to a mean of zero and a standard deviation of one.

### Exclusion Criteria

Parents reported their child’s DSM-V-based symptoms of alcohol and drug use disorders using a self-administered computerized version of the Kiddie Schedule of Affective Disorders and Schizophrenia Scale for School-Aged Children (KSADS-COMP), which is used to assess psychiatric disorders in children and adolescents, including drug use (Kaufman et al., 2021). Subjects with any reported substance use (N = 63) based on the KSADS-COMP parental report were excluded. This report did not address individuals' use of caffeine.

### Imaging

All structural MRI images were acquired on 3T (Siemens, Phillips, and GE) scanners with 1 mm isotropic T1-weighted structural images, using either a 32-channel head or 64-channel headandneck coil. Total intracranial volume and the volumes of six subcortical structures were extracted: thalamus, caudate, putamen, hippocampus, amygdala, and nucleus accumbens. As this was a pediatric population prone to excessive head motion in the scanner, prospective motion correction on the GE and Volumetric Navigators on the Siemens platforms were done to mitigate this concern by applying real-time motion detection and correction. The Multi-Modal Processing Stream software package, which includes FreeSurfer 5.3 (Fischl et al., 2004; Ségonne et al., 2004, 2007), was used to process MRI data. After the MRI images are corrected for distortions and head motion and cross-modality associations are performed, the cortical surface is reconstructed, and subcortical and white matter regions of the brain are segmented. Following MRI quality control procedures (Hagler et al., 2019), subcortical volumes (hippocampus, nucleus accumbens, putamen, thalamus, caudate, amygdala) were averaged across the left and right hemispheres. Total intracranial volume and the volumes of six subcortical structures were extracted: thalamus, caudate, putamen, hippocampus, amygdala, and nucleus accumbens. For each ROI, the effects of age, sex, total intracranial volume, and the first ten genetic principal components were removed using the modelr package in R (R version 4.3.2, http://www.rproject.org). Across ROI’s, variation in the principal components collectively explained up to 0.6% of the variance in each ROI (cortical surface area: 0.002, cortical thickness: 0.006, hippocampus: 0.003, thalamus: 0.006, caudate: 0.004, nucleus accumbens: 0.004, amygdala:

0.004, putamen: 0.004).

Volumes for subcortical structures, including the hippocampus, nucleus accumbens, putamen, thalamus, caudate, amygdala, were extracted from FreeSurfer's automatic segmentation process. The FreeSurfer pipeline automatically segments subcortical structures using a probabilistic atlas and applies deformable templates to delineate structures. The volume of each structure is calculated as the sum of the voxel-wise segmentations multiplied by voxel volume. FreeSurfer outputs were reviewed for errors, and manual corrections were applied as necessary. An overview of the quality control procedures for subcortical volumes is provided in the ABCD 5.0 Release Notes (https://wiki.abcdstudy.org/release-notes/).

Cortical surface area measures were derived from FreeSurfer's automated reconstruction pipeline. This pipeline constructs a surface mesh for each hemisphere of the brain, with the boundary between gray matter and white matter defined as the pial surface and the boundary between gray matter and cerebrospinal fluid defined as the gray-white matter boundary. Surface area was calculated as the sum of the areas of all triangles in the surface mesh. Quality control procedures for cortical surface area are outlined in the ABCD 5.0 Release Notes.

## Statistical analyses

The OpenMx 2.21.11 software package (M. C. Neale et al., 2016) in R 4.3.2 (R Development

Core Team, 2018) with the Constrained Sequential Optimal Linear and Nonlinear Programming (CSOLNP) optimizer was used to test basic assumptions of mean and variance homogeneity, calculate phenotypic and twin pair correlations and their 95% confidence intervals, and to fit univariate and multivariate genetically informative twin models(M. Neale & Cardon, 1992).

## Tests of mean and variance homogeneity

Mean and variance homogeneity within twin and sibling pairs, as well as across zygosity and sex, was tested (M. Neale & Cardon, 1992). Under the null hypothesis, when randomly sampling twins from the population, one can expect equal means and variances across and within MZ and DZ twins, assuming there are no sex differences in the variable of interest (Jinks & Fulker, 1970). Prior to estimating phenotypic and twin pair correlations and model fitting, we tested basic assumptions concerning mean and variance homogeneity by constraining mean and variance estimates to be equal within twin pairs, across zygosity and regular sib-pair groups, and across sex. Accepting the null hypotheses of neither mean nor variance differences across the MZ, DZ, and regular sib-pair groups indicates that the distributions of the data are consistent with several assumptions of the classical twin study. In particular, there is no evidence of sibling interaction, which would generate different total variances of MZ and DZ twins when some genetic variation is present. Departures from multivariate normality would also increase the likelihood that these equality tests would fail.

## Phenotypic twin pair correlations

Phenotypic and twin pair correlations were computed using full maximum likelihood in OpenMx (version 2.21.1)(Neale et al., 2016). Regarding twin pair correlations, if familial aggregation is entirely attributable to shared family environments, then monozygotic (MZ) and dizygotic (DZ) twin pair correlations will be statistically equal. In contrast, if familial aggregation is entirely attributable to shared additive (or non-additive) genetic factors, then DZ twin pair correlations will be ½ (or less) the size of their MZ twin pair counterparts.

## Univariate Analyses

In univariate analyses, the total variation in each ROI was estimated as the sum of additive genetic (A), shared or common environmental (C), and non-shared or unique (E) environmental variance components using an 'ACE' variance component model (Verhulst & Neale, see Fig. 1). Note that the non-shared environmental effects (E) are, by definition, uncorrelated and include measurement error. The association of variance with these sources exploits the expected genetic and environmental correlations between MZ and DZ twin pairs. MZ twin pairs are genetically almost identical, whereas DZ twin pairs share, on average, half of their genes. Under the equal environments assumption, the model assumes that shared environmental effects (C) are equal in MZ and DZ twin pairs, and that the magnitude of genetic and environmental effects is the same for both twins in a pair. Therefore, the MZ and DZ twin pair correlations ($r_A$) for additive genetic effects are fixed to 1.0 and 0.5, respectively, as opposed to the shared environmental twin pair correlation (rc), which is fixed to 1.0 regardless of zygosity. Given that non-twin sibling pairs also share, on average, half of their genes, we included non-twin sibling pairs as a separate group in addition to the DZ group after establishing mean and variance homogeneity. Note that since our analyses relied on a non-clinically ascertained community dwelling sample, we herein refer to all A, C, and E variance components as genetic and environmental 'influences.'These variance components assume any observed variation in normal development comprises both risk and protective factors.

This figure illustrates a classical twin design univariate analysis that decomposes variation in a brain region of interest (ROI) into additive genetic ($A_1$), shared environmental ($C_1$), and unique environmental ($E_1$) components. Single-headed arrows represent regressions, while double headed arrows represent correlations. The shared environmental correlation (rC) is fixed to 1.0 for both MZ and DZ twins, reflecting the equal environments assumption. Cortical SA = Total cortical surface area (example ROI), rMZ = Expected correlation for monozygotic twins (fixed to 1.0). rDZ = Expected correlation for dizygotic twins (fixed to 0.5). $A_1$ = Additive genetic factors for Twin 1 and Twin 2, $C_1$ = Shared environmental factors for Twin 1 and Twin 2. $E_1$ = unique environmental factors for Twin 1 and Twin 2. a11, c11, e11 represent the variance components for the latent factors representing the influence of $A_1$, $C_1$, and $E_1$ on the ROI.

## Multivariate analyses

The univariate model was extended to the multivariate case to estimate the size and significance of the impact of ROI-specific and drug-related PRSs to variance in each of the 8 ROIs. As illustrated in Fig. 2, the contributions of the SU/SUD and ROI-specific PRSs to the latent variance at each ROI are denoted by β31 and β21 pathway coefficients, respectively. To allow for background confounding, PRSs were also allowed to correlate via the a32 double-headed arrow. Variance in the latent ROI factor not captured by the two PRSs is explained by the residual additive genetic (A), common (C), and non-shared (E) environmental influences denoted by the a11, c11, and e11 variance components respectively. Finally, to scale the relative contribution of the PRSs, we estimated their standard deviations (δ11 & δ22) while constraining the latent PRS variances to one (Dolan et al., 2021). An equivalent approach would be to standardize the PRS data prior to analysis.

Multivariate twin model incorporating polygenic risk scores (PRSs) for brain structure and substance use. This figure illustrates a multivariate twin model that incorporates PRSs to examine the genetic and environmental influences on brain structure, using cortical surface area as an example. The model includes additive genetic (A), shared environmental (C), and unique environmental (E) influences on cortical surface area, as well as the effects of PRSs for cortical surface area (β21) and general liability to SU and SUD (SU/SUD; β31). The model allows for a correlation between the two PRSs (double-headed arrow between PRS variables). Each multivariate analysis followed a two-step approach: First, the best-fitting univariate model (ACE, AE, CE, or E) for each region of interest was determined; next, the significance of both the ROIspecific and the CUD/SUD PRSs was tested. This approach enabled us to test competing nested models representing alternative explanations for the sources of variance in brain structure, including models excluding either the SU/SUD PRS or the CUD PRS.

## Model Fit

In the univariate case, we determined the most likely sources of variance by fitting three additional sub-models in which the (i) C, (ii) A, and (iii) C and A influences were fixed to zero, i.e., we compared the likelihoods of the AE, CE, and E models, respectively. In the multivariate case, once the best fitting model was chosen, i.e., i) ACE, ii) AE, iii) CE, or iv) E, we then estimated the association of each ROI with the ROI PRS and the SU/SUD PRS, as illustrated by the β21 and β31 pathways coefficients, respectively (See Fig. 2). Models were compared using the change in the minus two Log-Likelihood (Δ-2LL) and Akaike's Information Criterion (AIC) which balances model fit against model complexity (Akaike, 1987).

The multivariate analysis was then re-run, substituting the CUD PRS for the SU/SUD PRS. The significances of the A, C, and E parameters, in the univariate and multivariate analyses and the β31 and β21 in the multivariate case were determined using the change in the minus two LogLikelihood (Δ-2LL). Under certain regularity conditions, this change is asymptotically distributed as a chi-square with the degrees of freedom equal to the difference in the number of free parameters in the two models (Steiger et al., 1985). The selection of the most suitable model used AIC (Akaike, 1987). In addition to examining the AIC, we looked at the difference in -2 loglikelihood (-2LL) between models. Model identification was confirmed for each model with the OpenMx utility mxCheckIdentification (Hunter et al., 2021). The significance of the β21 and β31 parameters in Fig. 2 was determined by examining the change in -2 loglikelihood (-2LL) by dropping β21 and β31.

For each ROI, we began by determining the most likely sources of variance by modeling all five sources of variation (A + C + E + 2 PRSs), followed by three nested sub-models in which the i) C, ii) A, and iii) C and A influences were successively fixed to zero. Based on the best-fitting ACE, AE, or CE model, we then dropped the β31 and β21 pathway coefficients to determine the significance of the impact of the PRSs on each ROI. This model-fitting strategy was applied to all eight ROIs separately, beginning with the CUD PRS. We repeated this model fitting strategy after substituting the CUD PRS for the SU/SUD PRS.

### Results

Our data comprised N = 222, N = 328, N = 387 monozygotic, dizygotic, and non-twin sibling pairs respectively with structural MRI and genotypic data (see Supplementary Table S1-S3). The regressions of each ROI on its corresponding ROI PRS are shown in Table 1. The beta estimates ranged from − 0.06 in the total cortical surface area to 0.18 for both the caudate and average cortical thickness (mean β = 0.13) indicating the effectiveness of the ROI PRS in predicting some variation in some ROIs.

Table 1
Linear Mixed-Effects Model Estimates and R2 Values between ROI-specific PRS and ROI.

| PRS | β | Std. Error | T-Statistic | Marginal $R^2$ | Conditional $R^2$ |
|---|---|---|---|---|---|
| Cortical Surface | -0.06 | 0.01 | -4.62 | 0.00 | 0.64 |
| Cortical Thickness | 0.18 | 0.01 | 13.47 | 0.03 | 0.48 |
| Hippocampus | 0.13 | 0.01 | 9.93 | 0.02 | 0.56 |
| Nucleus Accumbens | 0.10 | 0.01 | 7.65 | 0.01 | 0.49 |
| Thalamus | 0.11 | 0.01 | 8.14 | 0.01 | 0.57 |
| Putamen | 0.17 | 0.01 | 12.70 | 0.03 | 0.59 |
| Caudate | 0.18 | 0.01 | 13.66 | 0.03 | 0.58 |
| Amygdala | 0.10 | 0.01 | 7.07 | 0.01 | 0.53 |
| Note: Effects of age, sex, and top ten genetic principal components were removed from each ROI prior to model fitting | | | | | |

## Tests of mean and variance homogeneity

The assumptions of mean and variance homogeneity within twin and sibling pairs and across zygosity and sex were satisfied (see Supplementary Table S4), with one exception. We were unable to constrain the DZ and sibling covariances to be equal for cortical surface area, as the DZ covariance was slightly smaller (0.39) than the sibling covariance (0.43).

## Twin Pair Correlations

Monozygotic (MZ) and dizygotic (DZ) twin pair correlations for each ROI are shown in Supplementary Table S12.The average MZ twin pair correlation was 0.78 and ranged from 0.70 to 0.91. In contrast the average DZ twin pair correlation was 0.39 and ranged from 0.37 to 0.48. This pattern is broadly consistent with variance attributable to additive genetic and unique environmental influences, and with a prior report (Maes et al., 2023).

## Phenotypic correlations

Full information maximum likelihood pairwise phenotypic correlations (see Table 2) and their 95% confidence intervals are shown in Supplementary Tables 13–16. These statistics include correlations between the ROIs, with their corresponding ROI PRSs, the CUD PRS and the SU/SUD PRS. The within-region correlations between each ROI and its corresponding ROI-PRS ranged from − 0.06 to 0.18. The highest correlations were between the cortical surface area PRS and cortical surface area ($r = 0.19$, 95% CI: 0.17, 0.22) and cortical thickness PRS and cortical thickness ($r = 0.18$, 95% CI: 0.16, 0.21). The lowest correlations were between the amygdala PRS and amygdala ($r = 0.08$, 95% CI: 0.06, 0.11) and the thalamus PRS and thalamus ($r = 0.08$, 95% CI: 0.06,0.11). Correlations between the ROIs and the CUD PRS ranged from − 0.05 to 0.01.

The highest correlation was between CUD PRS and cortical surface area ($r = -0.05$, 95% CI: − 0.08, -0.02). Finally, correlations between the ROIs and the SU/SUD PRS ranged from − 0.01 to 0.01. Here, the highest correlation was between SU/SUD and caudate ($r = 0.01$, 95% CI: -0.01, 0.04).

## Univariate Model Fitting Comparisons

Full univariate model fitting comparisons are shown in Supplementary Table S6. For the putamen volume and cortical surface area, the AE model provided the best fit to the data as judged by the non-significant change in chi-square and lowest AIC values. Here, familial aggregation is consistent with additive genetic influences, which accounted for 88% and 75% of the total variance putamen and cortical surface area. Conversely, for six other ROI

(hippocampus, nucleus accumbens, amygdala, cortical thickness, caudate, thalamus), the full ACE model provided the best fit. In these cases, additive genetic factors accounted for 37–58% of the total variance, while shared environmental factors accounted for 18–21% of the total variance. These findings suggest that in each case, genetic factors play a predominant role in familial aggregation, with non-shared environmental influences (E) explaining the remaining (12–44%) variation.

## Multivariate Model Fitting Comparison

The multivariate analysis followed a sequential approach, although we note that alternatively, all variance components (A, C, and E) and both PRSs could have been fitted simultaneously. Our sequential approach first identified the best-fitting model (ACE, AE, CE, or E) for each ROI using comparative model fitting (see Supplementary Tables S8 and S10), followed by testing the significance of both the ROI-specific and either the CUD or SU/SUD PRSs within these bestfitting models. False discovery rate (FDR) within each PRS group (CUD & SUD PRS) and across the eight regions analyzed for each group was used to adjust for multiple testing (see Supplementary Tables S12 and S14). This sequential approach allowed us to quantify the extent to which predisposition to substance use measured by PRS contributes to the variation in brain morphometry.

## Models with SU/SUD PRS

Multivariate model fitting revealed that an AE model best fit all regions of interest (ROIs) except cortical surface area, which was best explained by an ACE model (see Supplementary Table S10). As summarized in Table 4, for the seven ROIs best fit by the AE model, additive genetic influences (A) explained 68–87% of the variance, with non-shared environmental factors (E) accounting for the remainder (0.09-29%). In the cortical surface area ACE model, additive genetic, shared environmental, and non-shared environmental factors were associated with 53%, 26%, and 18% of the variance, respectively.

We then tested the significance of each polygenic risk score (PRS) within these best-fitting models. The ROI-specific PRSs ($\beta_{21}$) significantly contributed to all ROIs, with standardized pathway coefficients ranging from 0.04 to 0.06, explaining 0.2–0.4% of the variance. It is important to note that these contributions from the ROI-specific PRSs are consistent across both the SU/SUD and CUD PRS models, as they represent the same PRSs predicting variances in the same ROIs.

Notably, the SU/SUD PRS (β31) significantly predicted variation in only one ROI: the nucleus accumbens (β31 = 0.05, 95% CI [0.00, 0.10]), though this effect was marginally significant. For the caudate, the SU/SUD PRS's impact was not significant (β31 = 0.04, 95% CI [-0.01, 0.09]). In the remaining six ROIs, including cortical surface area, the SU/SUD PRS did not significantly contribute to the variance. However, after FDR correction for multiple testing, these results were not significant.

## Models with CUD PRS

We first determined the best-fitting overall model for each region of interest (ROI). This revealed that an AE model best fit all ROIs except cortical surface area, which was best explained by an ACE model (see Supplementary Table S8). We then tested the significance of each PRS within these best-fitting models. As summarized in Table 3 (see Supplementary Table S12 for model fitting comparisons), the ROI-specific PRSs significantly contributed to all ROIs, with standardized pathway coefficients ranging from 0.09 to 0.21. For the seven ROIs best fit by the AE model, additive genetic influences explained 68–87% of the variance, with non-shared environmental factors accounting for the remainder (9–29%). In the cortical surface area ACE model, additive genetic, shared environmental, and non-shared environmental factors associated with 54%, 18%, and 18% of the variance, respectively. Notably, the CUD PRS did not significantly contribute to any ROI.

Table 3
Standardized variance components (95%CIs) attributable to additive genetic (A), common environment (C), and nonshared environmental (E) influences under each best fitting multivariate model.

| Region | A | C | E | β21- ROI-specific PRS | β31-CUD PRS |
|---|---|---|---|---|---|
| Cortical Surface Area | 0.56 (0.44, 0.67) | 0.26 (0.15, 0.36) | 0.18 (0.15 ,0.22) | 0.13 (0.08, 0.18) | - |
| Cortical Thickness | 0.72 (0.67, 0.77) | - | 0.27 (0.23, 0.33) | 0.20 (0.15, 0.24) | - |
| Amygdala | 0.73 (0.67, 0.77) | - | 0.27 (0.23, 0.32) | 0.10 (0.05, 0.15) | - |
| Hippocampus | 0.77 (0.73, 0.81) | - | 0.23 (0.19, 0.27) | 0.13 (0.09, 0.18) | - |
| Thalamus | 0.77 (0.72, 0.81) | - | 0.23 (0.19, 0.28) | 0.09 (0.04, 0.14) | - |
| Nucleus Accumbens | 0.71 (0.65, 0.76) | - | 0.29 (0.24, 0.35) | 0.09 (0.04, 0.14) | - |
| Putamen | 0.91 (0.81, 0.93) | - | 0.09 (0.07, 0.11) | 0.20 (0.15, 0.24) | - |
| Caudate | 0.83 (0.86, 0.88) | - | 0.14 (0.12, 0.17) | 0.21 (0.16, 0.26) | - |

Note: Each region of interest (ROI) adjusted for age, sex, total intracranial volume, and the top 10 genetic principal components. β21 = causal path from ROI-specific PRS to corresponding ROI, β31 = causal path from Cannabis Use Disorder (CUD) PRS to

ROI. Also shown are the standardized pathway coefficients of the ROI-specific and CUD PRSs.

Table 4
Summary of best fitting multivariate models testing the standardized relative contributions of additive genetic (A), common environment (C), and non-shared environmental (E) influences, and the ROI-specific and SU/SUD PRS.

| Region | A | C | E | β21- ROI-specific PRS | β31-SU/SUD PRS |
|---|---|---|---|---|---|
| Cortical Surface Area | 0.55 (0.44, 0.67) | 0.26 (0.16, 0.36) | 0.18 (0.15, 0.22) | 0.13 (0.08, 0.17) | 0.06 (0.01, 0.11) |
| Cortical Thickness | 0.72 (0.67, 0.77) | - | 0.27 (0.23, 0.32) | 0.20 (0.15, 0.24) | - |
| Amygdala | 0.72 (0.67, 0.77) | - | 0.27 (0.22, 0.33) | 0.20 (0.15, 0.24) | - |
| Hippocampus | 0.77 (0.672, 0.81) | - | 0.23 (0.19, 0.27) | 0.13 (0.09, 0.18) | - |
| Thalamus | 0.77 (0.72, 0.84) | - | 0.23 (0.19, 0.28) | 0.09 (0.04, 0.14) | - |
| Nucleus Accumbens | 0.70 (0.65, 0.81) | - | 0.29 (0.24, 0.34) | 0.09 (0.04, 0.14) | 0.05 (0.00, 0.10) |
| Putamen | 0.91(0.89, 0.93) | - | 0.09 (0.07, 0.11) | 0.20 (0.15, 0.24) | - |
| Caudate | 0.86 (0.83, 0.88) | - | 0.14 (0.12, 0.17) | 0.21 (0.16, 0.25) | 0.04 (-0.01, 0.09) |

Note: Each region of interest (ROI) adjusted for age, sex, total intracranial volume, and the top 10 genetic principal components. β21 = causal path from ROI-specific PRS to corresponding ROI, β31 = causal path from Cannabis Use Disorder (CUD) PRS to ROI. Lower bound CI for the General substance use/substance use disorder (SU/SUD) PRS contribution to Nucleus Accumbens marginally significant. β31 = 0.004, β31 = 0.002

### Discussion

We hypothesized that higher polygenic risk scores for CUD and SU/SUD would predict reduced volumes, surface area, and thickness in eight selected brain regions. Among the eight regions of interest explored, only total cortical surface area and nucleus accumbens volume were associated with substance use/substance use disorder polygenic risk scores in drug-naive adolescents. After FDR correction, only the association with cortical surface area remained significant, though with a very small effect size, and all other brain regions showed no significant associations. Genetic risk for CUD showed no significant associations with any of the eight regions of interest. These findings contribute to our understanding of the relationship between genetic risk for substance use and brain structure, although they cannot definitively determine causality. The limited associations in our drug-naive sample suggest that previously observed neuroanatomical differences in substance users might be more related to exposure than pre-existing variations in brain morphometry. However, a plausible alternative explanation is that SU-predisposing brain structure has not yet developed among these 9–10-year-old youth. Our findings suggest that brain morphometry at this age may not be a useful biomarker for identifying individuals at heightened genetic risk before substance use initiation. Future research should focus on other potential biomarkers or risk factors that might be more informative for early intervention strategies and employ methods that can more directly test causal relationships between genetic risk and brain structure.

Prior research based on adult and adolescent samples has found evidence of an association between brain structural differences and substance use (Chye et al., 2020; Gillespie et al., 2018; Lange et al., 2017; Miller et al., 2024; Paul & Bhattacharyya, 2018) and genetic associations with substance use and brain structures (Rabinowitz et al., 2022). Recent observations of brain structural differences between substance-naive youth who later initiate use versus those who do not (Miller et al., 2024) could reflect pre-existing genetic and environmental factors that simultaneously influence both brain development and substance use propensity rather than true temporal precedence. The genetically informative nature of our twin design provides stronger control over these potential confounding factors. Indeed, the current study investigated these associations in drug-naive adolescents while accounting for background genetic correlations between ROIs and substance use polygenic risk scores (PRS). Our analyses allowed these PRSs to correlate, thus accounting for their shared genetic influences. We found that the SU/SUD PRS predicted variation in only two ROIs and explained less than 1% of the total variance. These modest associations in drug-naive adolescents suggest that the more substantial brain structural differences observed in adult substance users may be more related to substance exposure than to pre-existing genetic risk factors, though future research using methods specifically designed to test causal relationships (such as Mendelian Randomization or causal modeling of the data from relatives) would be needed to support this hypothesis.

By analyzing heritability estimates from monozygotic (MZ), dizygotic (DZ), and non-twin sibling pairs, we found moderate to high heritability for cortical thickness and cortical surface area, with genetic factors accounting for 46% and 77% of the observed variation, respectively.

These findings are consistent with prior research, which showed that genetic factors contribute to 62% of the variation in cortical thickness at age 9 and 80% at age 12 (Teeuw et al., 2018).

However, previous studies have not controlled for the effects of total intracranial volume before analysis. Cortical surface estimates were slightly lower (77%) than earlier reports of 92% heritability in young adults (Ma et al., 2016). Our moderate to high heritability estimates in subcortical regions are similar to those found in previous research (Swagerman et al., 2014).

The associations between SU/SUD PRS and these ROIs are statistically significant but explain minimal variance in drug-naïve adolescents. However, these patterns may represent early indicators of emerging associations between genetic risk and brain structures implicated in addiction, which could potentially strengthen as individuals progress through adolescence. Considering that the SU/SUD PRS predicted < 1% of the variation in the cortical surface area and nucleus accumbens, these results suggest that genetic risk for substance use plays a minimal role in brain structure in drug-naïve adolescents. This could indicate either that genetic risk truly has limited influence on these structures at this developmental stage, or that current PRS measures are simply too weak to be used instruments for detecting such associations. Future improvements in the precision of the PRSs may prove more informative.

The nucleus accumbens, a key component of the mesocortical limbic system, has been extensively studied in addiction research (Volkow et al., 2007). While studies have demonstrated that substance use can lead to functional changes in the nucleus accumbens through dopaminergic mechanisms (Keiflin & Janak, 2015; Koob & Volkow, 2009), less is known about whether pre-existing structural variations might relate to genetic risk for substance use. Our findings suggest that, in drug-naïve adolescents, individual differences in this region are not associated with genetic risk influencing vulnerability to substance use and addictions, at least at this developmental stage.

The CUD PRS was not significantly associated with any brain regions, likely due to its limited precision as a genetic instrument. It explains only 0.04% of the variance in CU frequency based on GWAS summary statistics (Johnson et al., 2020). Consistent with this, Johnson et al. (2020) found no significant associations between the CUD PRS and three brain structures—total bilateral white matter volume, gray matter volume, and intracranial volume—in the ABCD dataset. Although a marginal association with white matter volume ($\beta = -0.04$; $p = 0.001$) was observed at a relaxed PRS p-value threshold ($\leq 0.5$), this explained only 0.18% of the variance, highlighting the weak predictive power of the CUD PRS for brain structure. Given that the CUD PRS explains only 0.04% of variance in CU frequency, the current analysis was likely underpowered to detect associations with brain structure. Advances in GWAS sample sizes and PRS methodology will be needed to effectively test for relationships between genetic liability to CUD and brain development.

To our knowledge, nearly all studies to date have not evaluated the validity of a prepositional model in which genetic risks for SU affect brain morphometry prior to SU or SUDs. We are aware of two studies whose results are consistent with such a model. Hatoum et al. (2023) found that genetic susceptibility to problematic alcohol use among drug-naive adolescents was linked to reduced volume in the left frontal pole and increased cortical thickness in the right supramarginal gyrus. Similarly, Rabinowitz et al. (2022) observed a positive association between genetic risk for alcohol use and greater surface area in the postcentral gyrus and cortical regions, as well as an association between a polygenic risk score (PRS) for regular smoking and multiple regions of interest (ROIs). After accounting for the correlation between the PRSs, correlated observations, and the joint impact of PRSs on brain morphometry, our study, in contrast, does not support the pre-dispositional role of genetic risk for SU/SUD or CUD on brain morphometry, despite finding a statistically significant but small impact of SU/SUD PRS on variation in 2 of 8 ROIs. It is, of course, plausible that genetic influences not directly identified as SU/SUD risk factors might correlate with brain morphometry in drug-naive subjects. These could potentially have indirect effects on SU/SUD susceptibility through complex pathways not captured in this study. In the meantime, our results are consistent with changes in brain morphometry arising as a consequence of SU and SUDs. They are equivocal, however, due to the possibility that brain structure has yet to develop to the point of associating with later substance use outcomes. Analyses of the ABCD sample's imaging of the brain at later ages will help to resolve this question.

The interpretation of our findings is significantly influenced by the strengths of the PRSs used in our study, particularly the CUD PRS. The varying predictive power of different substance use PRSs is reflected in recent Genome-Wide Association Studies (GWAS) results. The varying predictive power of different substance use PRSs aligns with recent GWAS (Hatoum et al., 2023, N = 1,025,550; Johnson et al., 2020, N = 384,925), such as Hatoum et al.'s trans-ancestral GWAS on problematic alcohol, tobacco, cannabis, and opioid use and Johnson et al.'s trans-ancestral GWAS meta-analysis of cannabis use disorder. Notably, the cannabis-dependent cases in the CUD GWAS were not restricted to non-smokers, which may have influenced the results. These findings align with previous studies examining the relationship between genetic risk and brain morphometry in drug-naive adolescents, highlighting the complexity of genetic influences on substance use disorders and brain structure.

It should also be noted that the current study controlled for total intracranial volume, in contrast to previous work (eg., Rabinowitz et al., 2022). This is important as cognitive ability and educational attainment have been strongly associated with increased total cortical surface area and intracranial volume (Cox et al., 2017; Nave et al., 2018). Conversely, cognitive ability and substance abuse phenotypes are negatively associated (Beverly et al., 2019; Gustavson et al., 2017; Schepis et al., 2018).

Finally, prefrontal, insula, and medial temporal cortical surface area and cortical thickness have also been shown to be significant mediators in the relationship between PRS for intelligence and general cognitive performance (Lett et al., 2019). Our results are in line with Wilson et al.'s (2018) prospective co-twin control study, which showed that differences in hippocampal volume between alcohol-using twins and non-using co-twins were more significant than the effects resulting from shared genetic and environmental liability toward problematic alcohol use, suggesting that any differences in hippocampal volume are likely due to alcohol exposure itself (Wilson et al., 2018).

## Limitations

These findings should be interpreted in the light of seven potential limitations. First, our inability to constrain DZ and sibling covariances for cortical surface area may have biased model estimates, potentially due to stochastic variation from our sample size, which larger samples could help clarify. In our analyses, regular sibling pairs showed higher similarity (covariance) in cortical surface area compared to DZ twins, and these covariances could not be constrained to be equal. Although twins typically share a unique pre- and postnatal environment, this effect might not be as strong in our sample, possibly due to stochastic variation due to our sample size. Typically, a higher DZ correlation compared to regular sibling pair correlation suggests the presence of a special twin environment effect (T) (M. Neale & Cardon, 1992). These effects may arise from the shared pre- and postnatal environments that twins experience due to being born and developing simultaneously. Sibling covariation is likely reduced relative to DZ twin pairs by not sharing the same environment at the same time: age by genotype or age by environment interactions are plausible mechanisms (Verhulst Eaves and Neale, 2014). In the current sample, these effects may be challenging to robustly detect or interpret with the sample size in the current dataset. For cortical surface area specifically, the inability to constrain DZ and sibling covariances to be equal suggests caution in interpreting those particular estimates, though this difference could reflect sampling variation rather than a true twin environment effect. In addition, this could bias the accuracy of the PRS predictions. While these covariance differences might reflect stochastic variation due to our sample size, larger samples would help clarify whether this apparent twin environment effect is robust and needs to be explicitly modeled. Future analyses combining ABCD with additional cohorts, such as the Queensland Twin Adolescent Brain Project (QTAB) (Strike et al., 2023), may allow the special twin environment or age by genotype interactions to be explicitly modeled.

The CUD PRS and brain structure PRSs currently explain a small variance in cannabis use and brain morphology in adults (Grasby et al., 2020; Johnson et al., 2020), warranting future reanalysis as predictive validity improves. Due to the low variance explained in adults, it's likely that PRSs derived from adult GWAS would account for even less variance in children. Including sex as a covariate adjusted for group mean differences but did not allow us to test sex-specific genetic effects. Additionally, regular sibling pairs had an average age gap of 1.3 years, which may have influenced results despite age correction, limiting exploration of age-related genetic and environmental heterogeneity. We employed a biometrical ACE model, assuming no dominance, epistasis, and GxE covariance or interaction. Examining broad brain regions may have obscured localized effects; for example, while the CUD PRS did not predict variation in our regions, prior studies have linked cannabis use to insula thickness changes (Chye et al., 2020; Rabinowitz et al., 2022), highlighting the need for finer-grained analysis. Lastly, as our sample included only European-descent individuals, these results may not generalize to other populations.

A further complexity with these analyses is that substance use is rarely limited to a single drug of abuse. Consequently, it is difficult to isolate, e.g., the effects of cannabis use from those of alcohol or tobacco use. Some of the earliest and most well-replicated studies of drug use on brain morphology include those of ventricle sizes, which are found to be higher among alcoholic than non-alcoholic study participants (Jernigan et al., 1982).

Finally, correcting the analyses for total intracranial volume may have substantially reduced associations between the ROIs and the PRSs. Results of not correcting are shown in the supplementary materials show that the significant associations between the ROIs and PRSs are no longer observed.

## Conclusion

To our knowledge, this is the first study to examine the relative contribution of a CUD or SU/SUD PRS to genetic variation in brain regions of interest in a genetically informative dataset. Our results suggest that variation in brain morphometry in baseline drug-naive subjects is unrelated to the genetic risk of CUD but is weakly related to general addiction and substance use risk (SU/SUD) in the nucleus accumbens volume and total cortical surface area. Considering that two out of eight brain regions were significantly predicted by SU/SUD risk, these results are consistent with the hypothesis that associations between substance use and brain morphometry seen in older samples are likely due to genetic risk shared between the substance use and the ROI, in addition to exposure to substance use. However, it is important to note that the phenotypic variance explained by the polygenic risk scores is very small. Future analyses should utilize longitudinal designs that examine brain morphometry before and after drug exposure. The ABCD Study® has great potential to do so, as long as the project stays funded at least through college years, which feature the highest rates of substance use.

## Declarations

Data Availability Statement

Data used in the preparation of this article were obtained from the Adolescent Brain Cognitive Development$^{SM}$ (ABCD) Study (https://abcdstudy.org), held in the NIMH Data Archive (NDA). The data that support the findings of this study are openly available in the NIMH Data Archive at https://nda.nih.gov/abcd. The authors confirm that the data supporting the findings of this study are available within the article and/or its supplementary materials.

**Author Information**

Virginia Institute for Psychiatric and Behavioral Genetics, Virginia Commonwealth University, 800 E. Leigh St., Richmond, Virginia 23298-0126

Sydney Kramer, Mei-Hsin Su, Mallory Stephenson, Luis F. S. Castro-de-Araujo, Yi Zhou, Michael C. Neale, Nathan Gillespie

University of Virginia, Department of Psychiatry and Neurobehavioral Sciences, 560 Ray C. Hunt Drive, Charlottesville, Virginia 22903

Roxann Roberson-Nay

Rutgers University, Department of Psychiatry, 671 Hoes Lane West, Piscataway, NJ, 08854

Jill A. Rabinowitz

Johns Hopkins Bloomberg School of Public Health, Johns Hopkins, Department of Mental Health, 615 N. Wolfe Street, Baltimore, MD 21205

Brion Maher

Contributions

MN and SK contributed to the study design, with NG leading the study design and formation.

Phenotype curation was completed by SK, MHS, MS, and YZ. LA and NG supervised analyses. SK and NG wrote the manuscript draft. JAR and BM provided feedback on the conceptual model and edited the manuscript. All authors provided edits to the manuscript.

**Corresponding Author**

Correspondence to Nathan Gillespie.

**Funding**

NG was supported by National Institute on Drug Abuse/NIH/DHHS (5R01DA052453-04)

**Conflict of Interest**

There are no conflicts of interest.

Ethical Approval

This study was approved by the local Institutional Review Board. Data used in the preparation of this article were obtained from the Adolescent Brain Cognitive Development (ABCD) Study (https://abcdstudy.org), held in the NIMH Data Archive (NDA). This is a multisite, longitudinal study designed to recruit more than 10,000 children age 9–10 and follow them over 10 years into early adulthood. The ABCD Study is supported by the National Institutes of Health and additional federal partners under award numbers U01DA041022, U01DA041028,

U01DA041048, U01DA041089, U01DA041106, U01DA041117, U01DA041120,

U01DA041134, U01DA041148, U01DA041156, U01DA041174,

U24DA041123, U24DA041147, U01DA041093, and U01DA041025. A full list of supporters is available athttps://abcdstudy.org/federal-partners.html.A listing of participating sites and a complete listing of the study investigators can be found athttps://abcdstudy.org/Consortium_Members.pdf.ABCD consortium investigators designed and implemented the study and/or provided data but did not necessarily participate in analysis or writing of this report. This manuscript reflects the views of the authors and may not reflect the opinions or views of the NIH or ABCD consortium investigators. The ABCD data repository grows and changes over time. The ABCD data used in this report came fromhttps://doi.org/10.15154/1523041.

**Consent to Participate**

All participants in ABCD provided informed consent (or assent).

**Consent for Publication**

N/A

**Acknowledgments**

We kindly thank all of the ABCD participants and their parents/guardians.

### Table 2

Table 2 is available in the Supplementary Files section.

### Figures

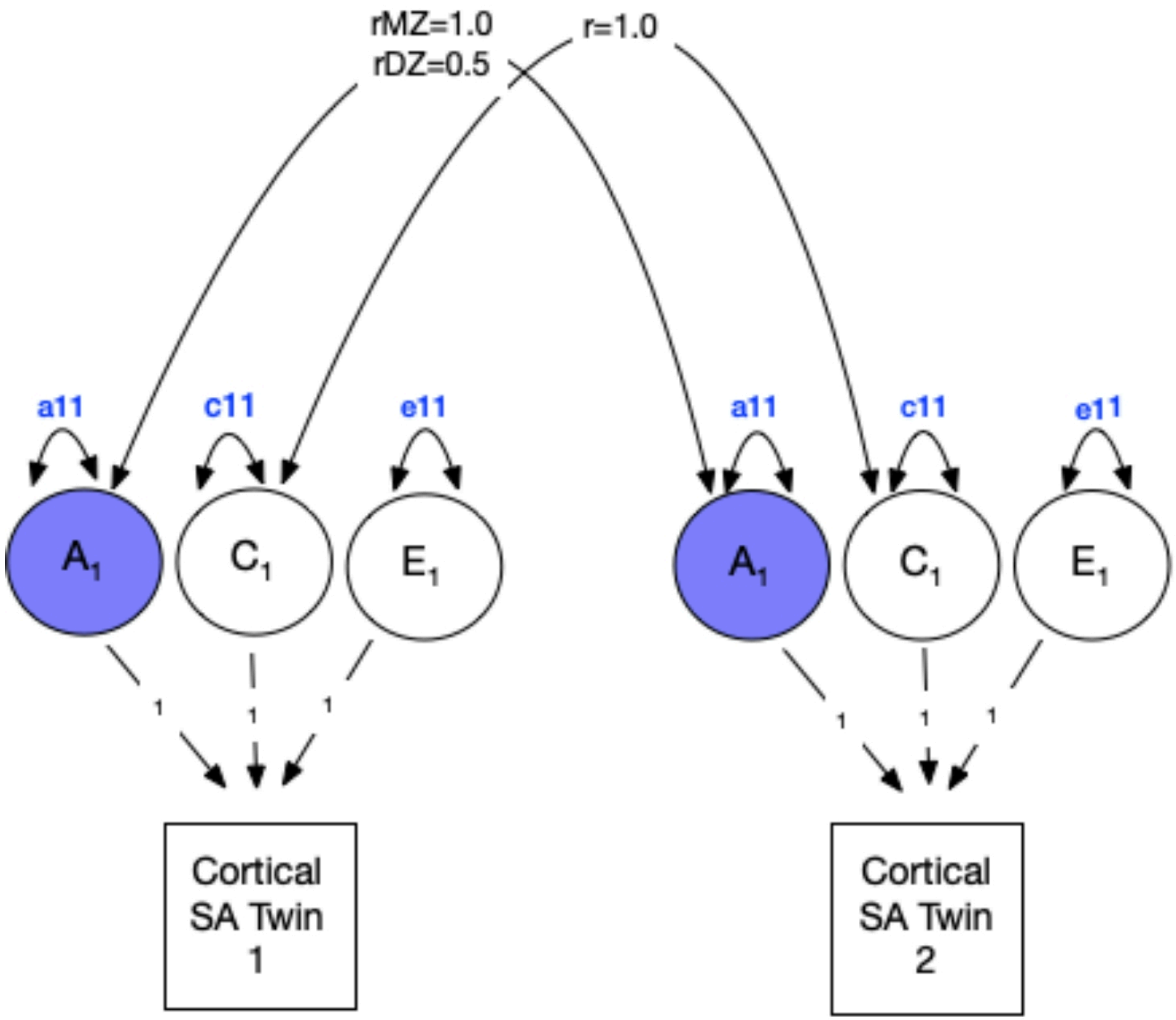

Figure 1

Theoretical univariate ACE twin model for brain structure

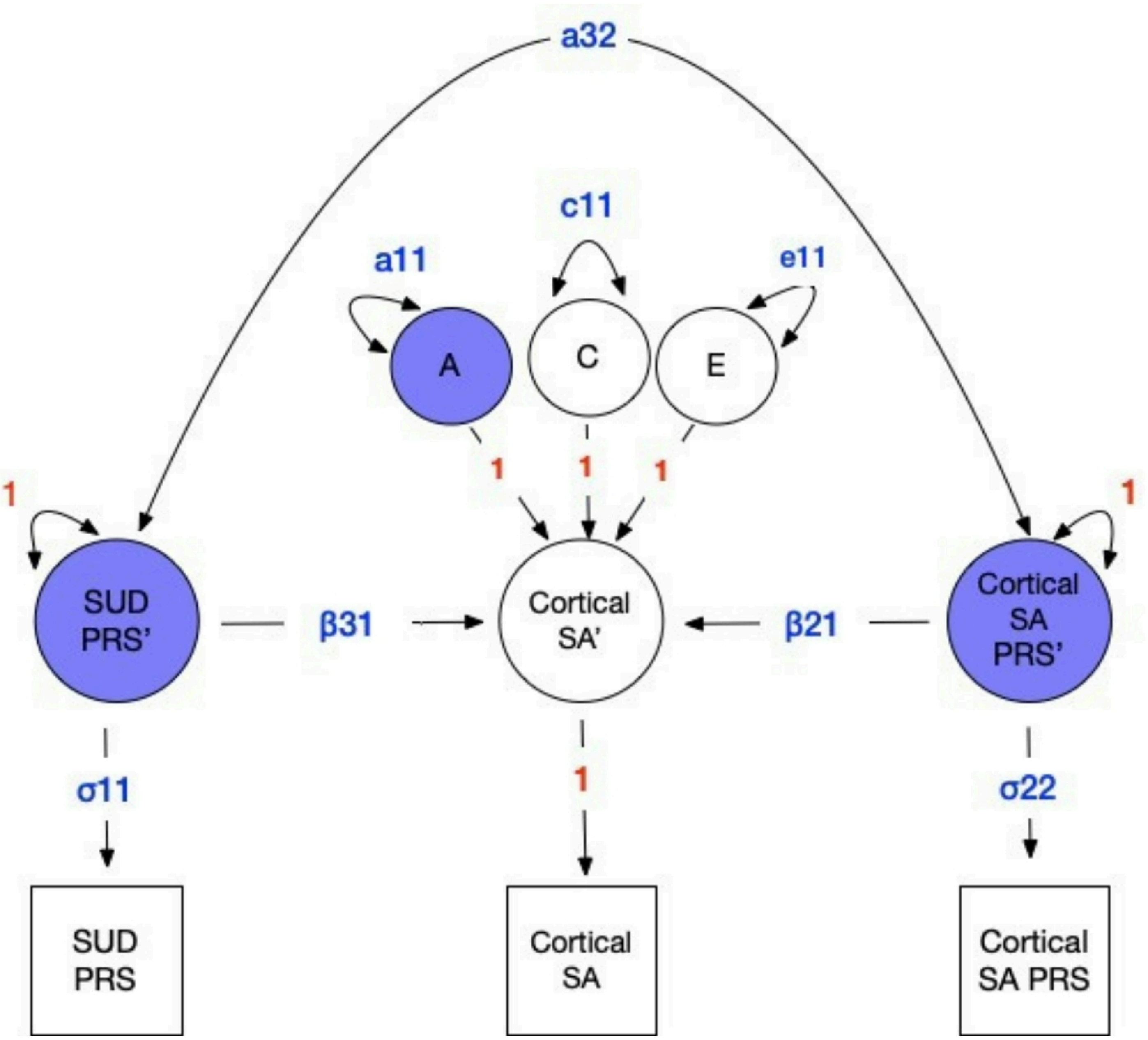

**Figure 2**

Multivariate twin model incorporating polygenic risk scores (PRSs) for brain structure and substance use

### Supplementary Files

This is a list of supplementary files associated with this preprint. Click to download.

- SupplementTablesABCDCUDSUD.xlsx
- Table2.docx
